# Straw Return and Tillage Regulate Soil N Pool via Modifying Soil Conditions and Bacterial Communities in Coastal Saline–Alkaline Land

**DOI:** 10.3390/microorganisms14061324

**Published:** 2026-06-12

**Authors:** Chunxiao Yu, Hanwen Liu, Shide Dong, Qian Ma, Haibo Zhang, Xiaoling Liu, Meicun Han, Shihong Yang, Guangmei Wang

**Affiliations:** 1 CAS Key Laboratory of Coastal Environmental Processes and Ecological Remediation, Yantai Institute of Coastal Zone Research, Chinese Academy of Sciences, Yantai 264003, China; cxyu@yic.ac.cn (C.Y.); hwliu@yic.ac.cn (H.L.); sddong@yic.ac.cn (S.D.); qma@yic.ac.cn (Q.M.); hbzhang@yic.ac.cn (H.Z.); xlliu@yic.ac.cn (X.L.); 2 Shandong Key Laboratory of Coastal Environmental Processes, Yantai 264003, China; 3 Yellow River Delta Saline-Alkali Agro-Ecosystem Observation and Research Station, Yantai Institute of Coastal Zone Research, Chinese Academy of Sciences, Dongying 257300, China; 4 Shandong Province Yellow River Delta Development Group Co., Ltd, Dongying 257347, China; hanmeicun@126.com; 5 College of Agricultural Science and Engineering, Hohai University, Nanjing 210024, China; ysh7731@hhu.edu.cn; 6 National Center of Technology Innovation for Comprehensive Utilization of Saline-Alkali Land, Dongying 257300, China

**Keywords:** bacterial community, organic nitrogen fractions, tillage practice, straw management, saline–alkaline soil

## Abstract

Straw return and tillage practices can alter the soil properties and regulate the bacteria communities, which mediate nitrogen (N) transformation and accumulation. This study aims to elucidate the mechanisms of microbially driven N retention, providing a foundation for soil management strategies. A field experiment was conducted in 2019–2022, six treatments were set up, including rotary tillage with/without straw (RTSR and RTNS), deep tillage with/without straw (DTSR and DTNS), subsoiling with/without straw (STSR and STNS). Soil properties, N pools/fractions and bacterial communities were measured. The results showed that straw return and tillage practices ameliorated soil environment (reducing bulk density (by 7–8% via DTSR and STSR) and salinity (with 57% and 26% increase in DTSR and STSR compared with RTSR, while rotary tillage significantly reduced salinity), increasing soil organic matter (via RTSR treatment, with 5–16% significant increase in two years) and effectively promoting N accumulation. The number of OTUs and the α-diversity significantly increased in 2022 compared with 2021. Specifically, tillage was the main driver of bacterial α-diversity, but there was no significant influence on bacterial β-diversity. Mental test results showed that N availability is a pivotal environmental factor shaping the bacteria α- and β-diversity. Structural equation modeling revealed that SON accumulation directly drove N accumulation via the “environmental improvement–specific microbial community structure” pathway. STSR is the optimal treatment for promoting N accumulation by maintaining active SON levels, which is an effective strategy for sustainable N management in the Yellow River Delta (YRD).

## 1. Introduction

Saline–alkali lands represent a widespread global agricultural challenge, as they severely limit crop productivity and sustainable land use [1]. Approximately 20% of the world’s agricultural land—around 1 billion hectares—is salt-affected [2,3]. The Yellow River Delta (YRD) of China is a typical region of coastal saline soil [4]. The utilization of saline–alkali land in China, particularly in the YRD, is essential for improving agricultural productivity and environmental sustainability, as it faces the dual constraints of high salinity–alkalinity and nutrient scarcity—particularly nitrogen (N), which is the primary limiting factor for crop growth.

Measures were taken to ameliorate saline–alkali soils, such as straw return and tillage. Straw return, strongly encouraged by the Chinese’s government to reduce N fertilizer input, not only improves soil properties (e.g., soil organic carbon (SOC) storage, Olsen P), especially in moderately saline–alkali soils [5,6,7], but also modulates soil N transformation process, including available N, fixed NH_4_^+^ and microbial N immobilization. Tillage practices, meanwhile, alter soil properties, aeration, and nutrient distribution [8,9]. For instance, rotary tillage enhances topsoil carbon (C) and N sequestration [8], deep tillage breaks plow pans to prevent upward salt migration, and subsoiling improves deep soil water permeability [10,11]. Straw return combined with tillage practices alters substrate input and soil microenvironments, thereby regulating nutrient transformation and cycling in soil–crop systems, and especially influences N retention and supply capacity [12,13]. Previous studies have reported that maize straw mulching with no tillage increases wheat yield by regulating root–soil interaction and N nutrition [13], which may be attributed to the reshaping of soil microbial community diversity, composition and assemblage, to change metabolic processes that influence soil quality [14,15]. However, most existing studies focus on the individual effects of straw return or tillage in non-saline soils, while their interactive impact on coastal saline–alkaline soils of YRD remain poorly understood. High soil pH and salinity reshape biogeochemical cycles [16,17,18]. Optimizing soil pH conditions can improve soil physical structure and enhance aggregate stability, thereby increasing soil C and N accumulation [17,19]. Specifically, it remains unclear how different tillage practices (rotary, deep tillage, subsoiling) synergize with straw return to modulate soil N transformation and accumulation in this typical saline–alkaline region.

Soil N accumulation in saline–alkaline soils is predominantly driven by soil organic nitrogen (SON), as approximately 90% of soil total N (TN) exists in organic forms [20,21]. Unlike TN, SON is a sensitive indicator of soil fertility, with its fractions (e.g., active soil organic nitrogen (ASON) and stable soil organic nitrogen (SSON)) directly determining crop N supply through microbial mineralization [22,23,24]. In saline–alkaline soils, SON turnover is constrained by salt stress, making it critical for clarifying how management practices regulate SON fractions [25]. Yet, existing studies prioritize total N, rather than focusing on SON fractions, failing to elucidate the core mechanisms underlying N retention in salt-affected soils under straw–tillage interactive conditions.

Microbial diversity is a key driver of N dynamics in agroecosystems [26]. However, the mechanisms by which tillage-straw interactions shifts microbial communities and subsequently regulate N accumulation remain insufficiently quantified, particularly in dynamic coastal saline–alkali regions. Previous studies have demonstrated that reduced tillage and straw amendment enhance microbial abundance by improving soil organic matter (SOM) and nutrient availability [5,27,28]. It has also been confirmed that maize straw return effectively modifies rhizosphere soil environment and microbial community under saline–alkaline stress [29]. Furthermore, microbial responses exhibit unique complexity due to salinity–alkalinity stress in saline–alkali soils. High soil pH and sodium content suppress nitrifier activity while favoring halotolerant taxa, leading to functional shifts in N cycling [16,30].

Soil bacterial communities are the core drivers of SON turnover and N transformation process [25]. In the YRD, saline–alkaline stress favors halotolerant taxa while suppressing nitrifiers [31], and the dominant bacterial phyla (e.g., *Pseudomonadota*, *Actinomycetota*, *Cyanobacteriota*) include functional groups responsible for SOM decomposition and N fixation [32]. However, current research focuses on microbial diversity directly mediate the N accumulation, but it remains unclear how straw–tillage practices altered functional bacterial communities to regulate SON fractions in saline–alkaline soils. To address these gaps, this study aimed to elucidate the key mechanisms of microbially driven N retention in coastal saline–alkaline soils. We hypothesize that (1) combined tillage and straw improves soil properties, thereby altering the distribution of SON fractions and promoting soil N accumulation; (2) the synergistic effect of straw return and tillage reshape soil bacterial community structure, especially changing microbial diversity; (3) different tillage depths (rotary, deep, subsoiling) interact with straw return to exert divergent effects on soil N accumulation, with variations linked to differences in the composition of functional bacterial communities and SON fraction dynamics. By investigating the effects of these practices, we aim to contribute to the development of sustainable N management strategies and the utilization of saline–alkali land in the YRD.

## 2. Materials and Methods

### 2.1. Site Description

**Figure 1:**
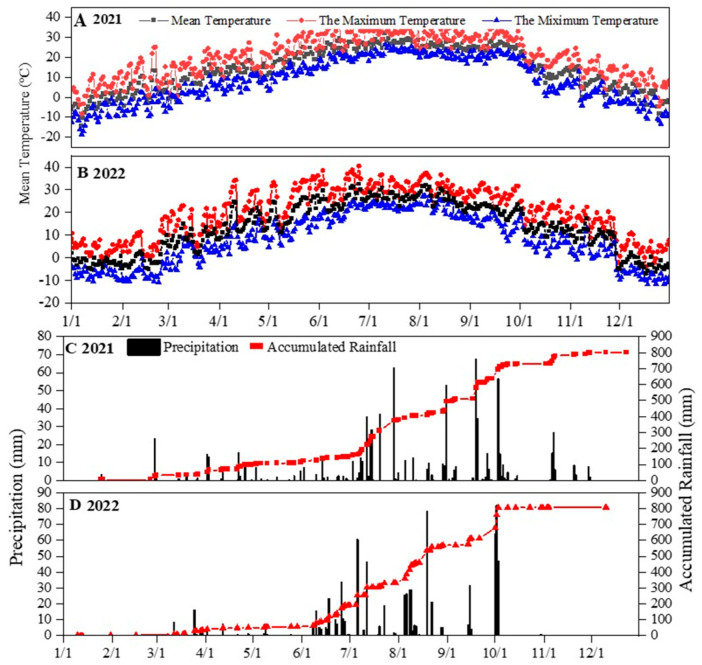
Detailed temperature and precipitation in 2021 and 2022. (**A**,**B**) represent the annual temperature in 2021 and 2022, while (**C**,**D**) indicate the annual precipitation in 2021 and 2022.

This study was conducted at the Yellow River Delta Saline-alkali Farmland Ecosystem Observation and Research Station of Yantai Institute of Coastal Zone Research, Chinese Academy of Sciences (37°18′ N, 118°39′ E). The basial properties of the experimental site was detected in 2019. The planting model was summer maize and winter wheat rotation. The soil type is classified as Fluvisol, with a silty loam texture (10.21% clay, 82.85% silt, and 6.94% sand). The salt content was 2–3‰, pH was between 8.3 and 8.5, SOM was 9.8–11.49 g/kg, TN was 0.61–0.88 g/kg, total phosphorus content (TP) was 0.56–0.82 g/kg, inorganic nitrogen content (IN) was 26.8–34.4 mg/kg. The valid phosphorus content (AP) was 12.46–18.13 mg/kg. Meteorological data (temperature and precipitation) during the experimental period are presented in Figure 1.

### 2.2. Experimental Design

The platform was initially established in 2019. Considering the feasibility of straw returning and tillage practices, a two-factor experiment was designed, and three tillage practices and two straw return methods were set up: rotary tillage (0–15 cm) + no straw (RTNS), rotary tillage (0–15 cm) + straw return evenly (RTSR), deep tillage (0–20 cm) + no straw (DTNS), deep tillage (0–20 cm) + straw return evenly (DTSR), subsoiling (0–40 cm) + no straw (STNS), subsoiling (0–40 cm) + straw return evenly (STSR). A total of 18 plots were set up, with six treatments and three replicates for each treatment, and the plot was 12 m × 7.5 m = 90 m^2^.

**Figure 2:**
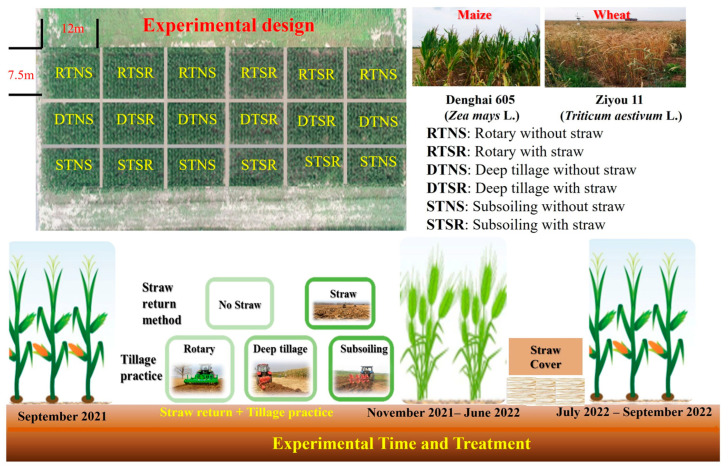
The experimental design and treatment.

The field adopted a typical double-cropping rotation system of winter wheat Ziyou 11 (*Triticum aestivum* L.) and summer maize Denghai 605 (*Zea mays* L.). Summer maize was sown in June and harvested in October, and maize yield measurement and straw direct return were carried out. The maize straw was fully returned (about 13.34 t ha^−1^). Each year, for maize cultivation, 225 kg N, 90 kg P_2_O_5_, and 90 kg K_2_O were applied per hectare as basal fertilizers. Winter wheat was sown in October and harvested at June, with 240 kg N, 150 kg P_2_O_5_ and 105 kg K_2_O applied per hectare as basal fertilizers. When crop grain was harvested, the straw (about 10 t ha^−1^) was sliced into 5–10 cm sections using a residue chopper and scattered on the surface soil. Wheat straw was returned to the field through the surface mulching method, and summer corn was directly sown with fertilizer without tilling. Weeding, pesticide application and irrigation are the same as the regular management measures. The detailed information is presented in Figure 2.

### 2.3. Soil Sampling

Soil sampling was conducted on 12 October 2021, and 8 October 2022. Eighteen soil samples were collected from the 0–20 cm soil layer in each plot using a 3 cm diameter auger via the five-point method. Before sample processing, visible plant roots and residues were manually removed from all soil samples. The cores from the same plot were pooled, homogenized, and sieved through a 2 mm mesh. Subsequently, each composite sample was subdivided into three portions: the majority was refrigerated at 4 °C for immediate use, a subset was cryopreserved at −80 °C for microbial community analysis, and a 150 g aliquot was air-dried for chemical characterization, including pH, electrical conductivity (EC), TN, SOM and SON fractions.

### 2.4. Soil Properties Analysis

Soil pH and EC were conducted at a 1:5 soil—water ratio. Soil bulk density (BD) was detected through the cutting ring method. Soil ammonia nitrogen (NH_4_^+^-N) and nitrate (NO_3_^−^-N) were extracted with 2 M KCl and analyzed using the continuous flow analyzer (AA3, Germany). Soil inorganic nitrogen was the sum of soil NH_4_^+^-N and NO_3_^−^-N. The partial apparent nitrification rate (ANR) was calculated as NO_3_^−^-N divided by inorganic nitrogen (IN). The acid hydrolysis method was employed to determine soil organic nitrogen (SON) fractions. Soil TN was determined with an element analyzer (Vario Macro cube, Elementar, Germany). SOM was analyzed using the dichromate oxidation method. Soil active SON (ASON) was the sum of hydrolysable NH_4_^+^-N + amino acid N + amino sugar N, and stable SON (SSON) was the sum of hydrolysable unknown N + acid-insoluble N [19].

### 2.5. Amplicon Sequencing

Microbial DNA was extracted from three replicates of each sample with 0.5 g soil. DNA quality and concentration were determined through 1.0% agarose gel electrophoresis and a Nano Drop 2000 spectrophotometer (Thermo Scientific, Wilmington, DE, USA) and subsequently stored at −80 °C prior to further use. The V4-V5 hypervariable region of the bacterial 16S rRNA gene was amplified using primer pairs 515F and 907R (5′-GTGCCAGCMGCCGCGG-3′ and 5′-CCGTCAATTCMTTTRAGTTT-3′) with a T100 Thermal Cycler PCR thermocycler (Bio-Rad, Hercules, CA, USA) [33]. Each 20 µL PCR reaction mixture contained 4 μL of 5 × Fast Pfu buffer, 2 μL 2.5 mM dNTPs, 0.8 μL primer, 0.4 μL Fast Pfu polymerase, and 10 ng of template DNA, made up to volume with H_2_O. To detect any potential contamination during the amplification process, a negative control (using ddH_2_O as template) was included in each PCR run. The PCR amplification cycling conditions included: 95 °C for 3 min, 27 cycles of denaturing at 95 °C for 30 s, annealing at 55 °C for 30 s and ending at 72 °C for 45 s, and single extension at 72 °C for 10 min and end at 4 °C. Amplified products were extracted from 2% agarose gel and purified with a PCR Clean-Up Kit (YuHua, Shanghai, China) according to the manufacturer’s instructions and quantified using Qubit 4.0 (Thermo Fisher Scientific, Wilmington, DE, USA). The purified amplicons were then pooled in equimolar concentrations. Sequencing was commissioned to Shanghai Majorbio Bio-technology Co., Ltd., (Shanghai, China) which was performed on the Illumina MiSeq sequencing platform (Omega Bio-tek, Norcross, GA, USA), using a 2 × 250 bp paired-end sequencing mode for high-throughput sequencing.

### 2.6. Statistical Analyses

Soil data were presented as the average of three replicates. All statistical analyses mentioned above were performed using SPSS 16.0 (SPSS, Chicago, IL, USA). The physicochemical properties and soil N indicators of each treatment were all three means and standard deviations. Multi-factor analysis of variance (ANOVA) and Duncan’s multiple comparisons test were used to analyze significant differences (*n* = 3). Bioinformatic analysis of the soil was conducted via the Majorbio Cloud platform (https://cloud.majorbio.com). Optimized sequences were clustered into OTUs using UPARSE v7.1 (97% similarity). Chimeric sequences were identified and removed during this process. To ensure comparability with previous studies and given that the 97% similarity threshold is sufficient to capture the main differences in the targeted communities (provide a brief justification if needed), we opted for OTU-based analysis rather than amplicon sequence variants (ASVs). The data underwent log transformation and were centered by species for principal component analysis (PCA), utilizing OTU information, rarefaction curves and alpha diversity (Chao richness, Shannon index) to measure species differences. The similarity between microbial communities in different treatments was gauged through PCA based on Bray–Curtis’s distance. IBM SPSS Amos 28 Graphic software facilitated the creation of a structural equation model to discern the primary drivers of N fixation in soil. Structural equation model (SEMs) was conducted independently in different periods of 2021 and 2022 to ensure the linearity in the relationships between different variables. The model’s fit was assessed by several metrics: Chi-square maximum likelihood test, Chi-square degree of freedom (CMIN/DF), goodness-of-fit index (GFI), comparative fit index (CFI), and approximate root mean square error (RMSEA). A non-significant Chi-square test (*p* > 0.05) suggested an acceptable model [34].

## 3. Results

### 3.1. Soil Physical and Chemical Indexes and N Distribution

Soil physicochemical properties are presented in Table 1. In 2022, compared with RTSR treatment, DTNS and DTSR significantly reduced soil pH (*p* < 0.05); rotary tillage (RTNS and RTSR) significantly reduced soil EC, compared with deep tillage and subsoiling (*p* < 0.05). In addition, compared with RTSR, DTSR and STSR significantly increased soil EC about 57% and 26%, respectively (*p* < 0.05), and DTSR significantly increased soil EC about 9% compared with DTNS (*p* < 0.05). Although this difference was not significant in 2021, it demonstrates that the effects of tillage and straw incorporation on soil pH and EC became statistically significant in 2022. Specifically, rotary tillage decreased soil EC, while deep tillage or subsoiling combined with straw significantly increased soil EC. RTSR facilitated SOM accumulation in both years, with SOM content significantly higher compared with RTNS (5% and 16%, *p* < 0.05), which indicates that straw return is the core factor for increasing the organic matter in saline–alkali land soil. In 2021, deep tillage and subsoiling both reduced soil BD by 1–7%, but increased it by 3–6%. While in 2022, DTSR and STSR significantly reduced soil BD (8%, 7%) compared with RTSR. This result suggested that DTSR and STSR can effectively improve the physical structure of saline–alkali land in the YRD. Additionally, DTSR significantly lowered soil TN, IN and SON contents than STSR (*p* < 0.05). Multi-way ANOVA revealed that soil EC was significantly affected by tillage practices, year and the interaction of straw × tillage, tillage × year (Table 1, *p*  <  0.05). Both year and the straw × tillage interaction exerted significant effects on soil IN, SON and TN (Table 1, *p*  <  0.05).

The variations in SON fractions are listed in Table 2. In 2021, DTNS and DTSR increased the amino acid N, but decreased the hydrolysable unknown N. In 2022, DTSR reduced the active SON components (hydrolysable NH_4_^+^-N, amino acid N, and amino sugar N), and increased stable SON (hydrolysable unknown N and acid-insoluble N). In addition, DTNS, DTSR, STNS and STSR treatments significantly reduced stable SON relative to RTNS and RTSR (Table 2, *p*  <  0.05). Multi-way ANOVA indicated tillage practice, year and their interactions significantly affected the amino acid N, amino sugar N, hydrolysable unknown N, acid-insoluble N and stable SON (Table 2, *p*  <  0.05). Moreover, straw return and its interactions with tillage and year also significantly affected soil amino acid N and stable SON (Table 2, *p*  <  0.05). Collectively, straw return played a significant role in saline–alkali soil improvement: rotary/deep tillage with straw return favored SOM accumulation; deep tillage with straw return improved soil structure; and subsoiling with straw return promoted N transformation and accumulation.

### 3.2. Effects on Bacterial Community Diversity

#### 3.2.1. Microbial Composition Analysis

**Figure 3:**
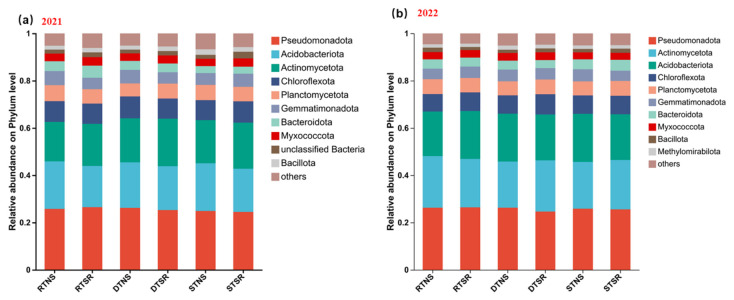
The relative abundance of species at the phylum level in 2021 and 2022. (**a**) Relative abundance in 2021; (**b**) relative abundance in 2022. Calculate the average value of the grouped samples, and combine the samples with <10 abundances as others.

**Figure 4:**
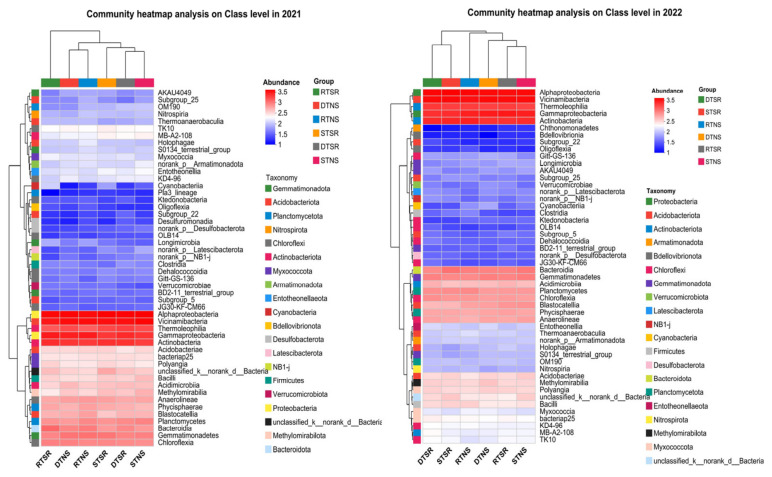
Relative abundance of microbial communities at the class taxonomic level. The top 50 species with the highest total abundance were selected, and the mean abundance was calculated for each group of samples (*n* = 3). Hierarchical clustering of species and samples was conducted using the average linkage method. All data were normalized with log_10_ transformation. The horizontal axis was labeled with sample or group names, and the vertical axis was labeled with species names. The abundance variations in different species in the samples were presented by the color gradient of blocks, and the values corresponding to the color gradient were displayed on the right side of the figure.

Bacteria communities in phylum and class level are showed in Figure 3 and Figure 4. The results indicated that tillage practices slightly altered bacterial community structure at both phylum and class levels (Figure 3 and Figure 4). *Pseudomonadota*, *Actinomycetota*, *Acidobacteriota*, and *Chloroflexota* were the predominant bacterial phyla across all treatments and years, accounting for more than 70% in 2021 and over 74% in 2022 (Figure 3). These key phyla primarily regulate soil C and N cycling. In 2021, straw return showed higher *Acidobacteriota*, *Actinomycetota*, *Myxococcota* abundance than no straw (RTNS vs. RTSR, DTNS vs. DTRSR STNS vs. STSR), indicating straw input promoted decomposers, with minor tillage effects (Figure 3a). and the *Bacteroidota* OUT numbers (DTNS: 74, DTSR: 102, STNS: 257, STSR: 256) were significantly decreased compared to RTNS (908 OUTs) (*p* < 0.05), indicating that the bacterial community underwent rapid early succession, with tillage-induced soil disturbance acting as the core driver of community structural variation. In 2022, microbial responses varied across tillage regimes: subsoiling enriched *Bacteroidota* (6–22%) compared with rotary, RTSR favored *Actinomycetota* (7%) compared with RTNS, and DTSR decreased *Bacteroidota* (10%) compared with DTNS, though all these differences were non-significant (Figure 3b). Year-to-year comparison revealed that *Actinomycetota* replaced *Acidobacteriota* as the second most dominant phylum in 2022.

Hierarchical clustering analysis further revealed distinct temporal patterns of community differentiation. In 2021, tillage regime (RT/DT/ST) was the primary driver of bacterial community differentiation, while straw return exerted negligible effects within the same tillage practice. However, in 2022, straw-amended treatments (STSR and DTSR) formed distinct clusters, indicating that the effect of straw return on the bacterial community became more pronounced with increasing experimental duration, which is consistent with Figure 3. Overall, treatment-induced community dissimilarities were mainly reflected in gradient changes in relative abundance rather than complete separation. Specifically, in 2022, rotary tillage enriched class *Bacteroidia* and phylum *Bacteroidota* (Figure 3b and Figure 4), reflecting its preference for readily decomposable organic substrates in topsoil, whereas STSR/DTSR harbored higher abundances of *Actinobacteria*, *Thermoleophilia*, and *Vicinamibacteria*, which corresponded to the expected effects of straw return in promoting organic matter decomposition and improving the soil environment. Overall, short-term bacterial community succession was primarily driven by tillage disturbance, while straw return functioned as a cumulative regulator that became more prominent in the second year of the experiment.

#### 3.2.2. Relationships Between Samples and Species

**Figure 5:**
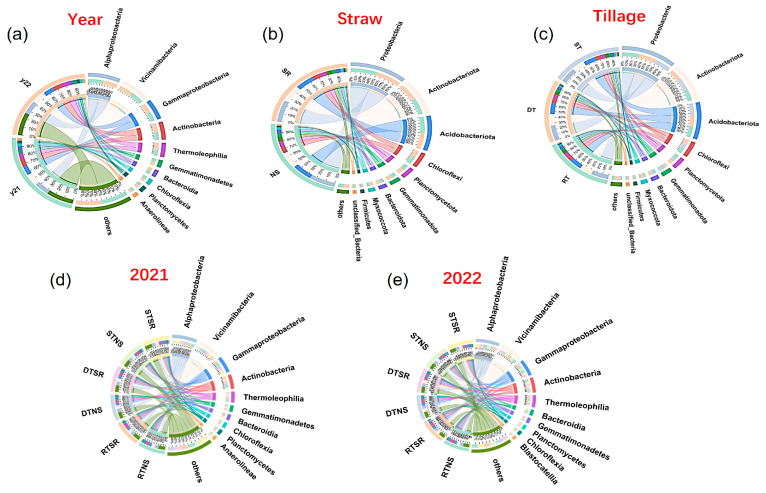
Circos diagrams of the relationship between treatments and classes. (**a**) Diagram of the two years, (**b**) diagram of straw return method, (**c**) diagram of three tillage practices, (**d**,**e**) diagram of different treatments in 2021 and 2022, respectively. Mean values were calculated for grouped samples (*n* = 3). Species with abundance less than 10 were merged into others. The left small semicircle depicts species composition of samples. Outer ribbons are colored by groups, and inner ribbons represent species, with ribbon lengths indicating relative abundance. The right large semicircle illustrates species distribution proportions across samples at the corresponding taxonomic level. Outer ribbons denote species, inner ribbons correspond to groups, and ribbon lengths reflect distribution ratios. y22: year of 2022; y21: year of 2021; NS: no straw return; SR: straw return; RT: rotary tillage; DT: deep tillage; ST: subsoiling.

Circos diagrams illustrated microbial species distribution across samples (Figure 5). Compared with 2021, the relative abundance of dominant phyla (*Vicinamibacteria*, *Gammaproteobacteria*, *Actinobacteria*, *Thermoleophilia*, *Bacteroidia*) increased 2–8% in 2022 (Figure 5d,e). Functionally, these microbes are associated with nutrient turnover, N fixation, organic matter degradation, and plant growth promotion, thereby enhancing soil ecological stability and fertility. Straw return increased the relative abundances of *Alphaproteobacteria*, *Actinobacteria*, *Thermoleophilia*, and *Chloroflexia* by 24%, while decreasing *Vicinamibacteria*, *Gammaproteobacteria*, and other species by about 2% (Figure 5b), thereby promoting carbon-degrading microbes and accelerating organic matter transformation and nutrient release. Tillage practices differentially influenced functionally distinct microbial taxa by altering soil structure: rotary tillage favored *Gammaproteobacteria*, *Actinobacteria*, and *Bacteroidia*, which enhanced rapid surface decomposition but potentially reduced community stability; deep tillage favored *Alphaproteobacteria*, *Thermoleophilia*, and *Gemmatimonadetes*, stimulating C and P cycling in deep soil; subsoiling enriched *Alphaproteobacteria* and *Vicinamibacteria*, supporting symbiotic N-fixing and structurally stable bacterial communities (Figure 5c).

#### 3.2.3. Microbial Community—α-Diversity

**Figure 6:**
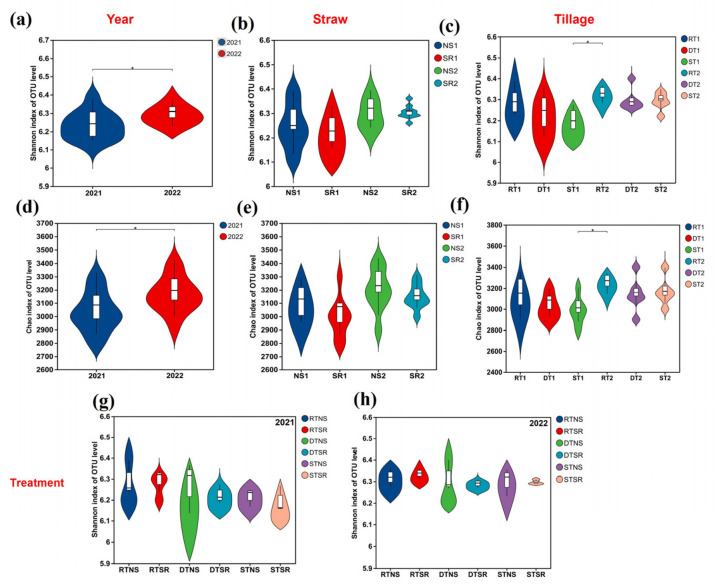
The impact of straw return and tillage practices on bacterial community α-diversity in 2021 and 2022 in OTU level. The numbers 1 and 2 (**b**,**c**,**e**,**f**) represent the years 2021 and 2022. (**a**,**d**) Shannon and Chao index of tillage practice. (**b**,**e**) Shannon and Chao index of straw return method. (**c**,**f**) Shannon and Chao index of years. (**g**,**h**) The Shannon diversity of different treatment influenced by tillage and straw in 2021 and 2022, respectively. Statistical analysis was performed using one-way ANOVA with FDR multiple testing correction; * represent significance of difference at the 0.05 level (Duncan). NS: no straw return; SR: straw return; RT: rotary tillage; DT: deep tillage; ST: subsoiling. The numbers 1 and 2 represent year of 2021 and 2022.

A total of 382,006 and 397,860 effective sequences were obtained in 2021 and 2022, respectively. The number of OTUs significantly increased in 2022 relative to 2021. OTU composition analysis revealed that two years of straw return and tillage significantly enhanced the species richness and distribution (*p* < 0.05, Figure 6a,d). However, the straw return method had no significant effect on bacterial diversity (Figure 6b,e). When tillage shifted from subsoiling in 2021 to rotary in 2022, treatment duration significantly affected bacterial diversity (Shannon and Chao indices) (Figure 6c,f). In 2021, tillage practices dominated Shannon diversity of soil bacterial, with RT  >  DT  >  ST (Figure 6g), indicating deeper tillage reduced microbial community richness and evenness. In 2022, the Shannon index stabilized, reflecting the convergence of community diversity over time (Figure 6h). These results demonstrate that tillage depth initially exerts a stronger influence on soil bacterial diversity than straw return, but the microbial community stabilizes with prolonged management.

### 3.3. Microbial Community—β-Diversity

**Figure 7:**
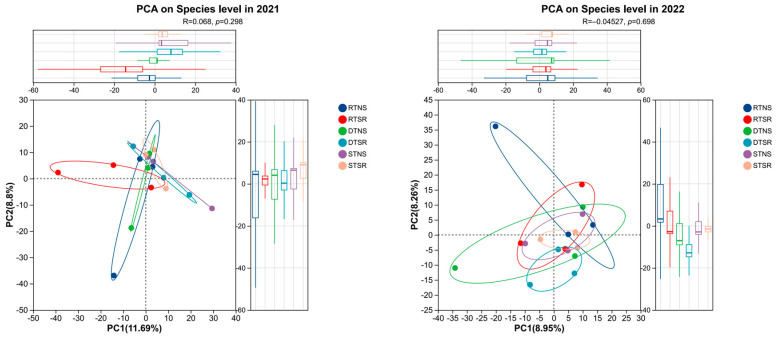
Principal component analysis based on bacterial community composition at the species level. Significance of difference at the 0.05 level. The analysis of similarities (ANOSIM) was used to compare the intergroup differences in species abundance, and Z-Score standardization was applied to environmental factors.

Principal component analysis (PCA) was performed to evaluate the effects of soil physicochemical properties and soil N allocation on soil bacterial species under different straw return and tillage regimes (Figure 7). The first two PCA axes explained 20.49% and 17.21% of microbial β-diversity in 2021 and 2022, respectively. No significant community separation was detected across treatments (ANOSIM, *p* > 0.05, Figure 7). In 2021, RTNS and RTSR were significantly separated along PC1, while in 2022, RTNS and DTNS showed significant separation. The PCA results revealed that tillage was the dominant driver of microbial community divergence, while straw return acted as an important stabilizing factor. Rotary and deep tillage, particularly without straw return, increased microbial community instability and heterogeneity. In contrast, straw return mitigated this disturbance, promoting the homogenization and stabilization of microbial community composition.

### 3.4. Correlation Between Bacterial Community and Environmental Factors

**Figure 8:**
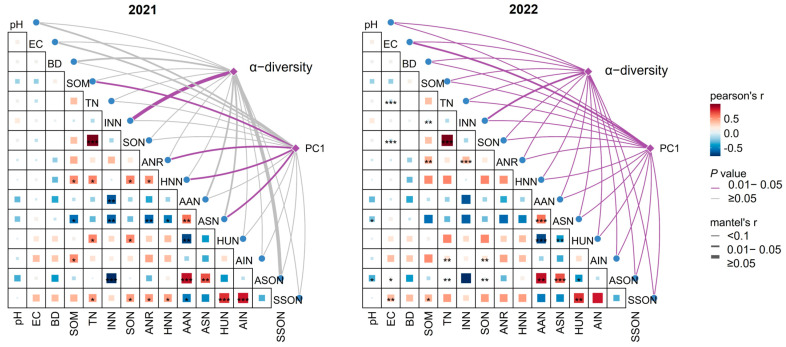
The Mantel test between environmental factors and microbial diversity. Line width is proportional to the Mantel’s R statistic, and line color denotes statistical significance. Pairwise comparisons of environmental factors are also shown, with color gradient and square size denoting Pearson’s correlation coefficient (*p* < 0.05). *, ** and *** indicate significant differences at the levels of *p* < 0.05, *p* < 0.01 and *p* < 0.001, respectively. PC1 is the first principal coordinate axis in the PCA result. EC: electrical conductivity; BD: soil bulk density; TN: soil total nitrogen; SOM: soil organic matter; INN: inorganic nitrogen; HNN: hydrolysable NH_4_^+^-N; AAN: amino acid N; ASN: amino sugar N; HUN: hydrolysable unknown N; AIN: acid-insoluble N. ASON: active SON; SSON: stable SON.

A Mantel test was conducted to explore the relationship between environmental factors and bacterial diversity (Figure 8). In 2021, bacteria α-diversity showed a highly significant positive correlation with IN (*p* < 0.05), and bacteria β-diversity was positively correlated with SOM, ANR, HNN, and ASN (*p* < 0.05). In 2022, bacteria α-diversity and PC1 were significantly correlated with the soil properties and N fractions (*p* < 0.05). These findings suggest that N availability is a pivotal environmental factor shaping the composition of bacterial communities in saline–alkali lands, and straw returning and tillage practices regulate N cycling, thereby fostering diverse and functionally active microbial communities. Increased availability of labile N pools (such as IN and amino acid N) drove a shift bacterial community structure trophic strategy from eutrophic to oligotrophic taxa. This transition ultimately enriched microbial taxa capable of metabolizing labile SOM and inorganic nitrogen substrates. In conclusion, the effects of management practices on soil microbiota were multifaceted and time-cumulative. These practices improved soil N availability and microenvironmental conditions, further regulating microbial ecological functions in saline–alkali soil ecosystems.

**Figure 9:**
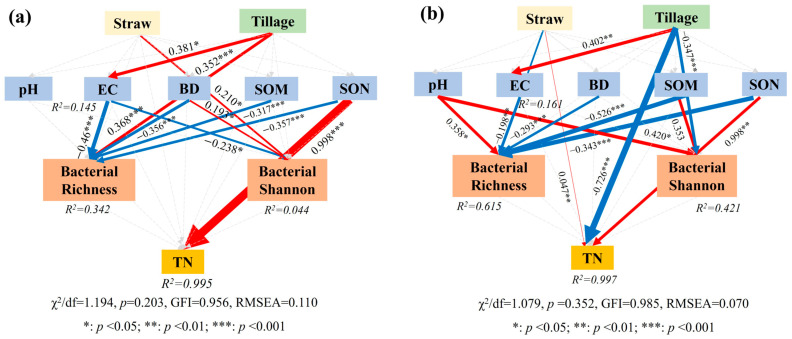
Structural equation models revealing the relationships among soil chemical properties and soil bacterial traits in 2021 (**a**) and 2022 (**b**). The dotted line represents the preset route, solid red and bold arrows indicate a positive correlation, while blue and bold arrows indicate a negative relationship. The arrow width is proportional to the strength of the path coefficients. R^2^ denotes the proportion of variance explained by the model. *, ** and *** indicate *p* < 0.05, *p* < 0.01 and *p* < 0.001, respectively. EC: electrical conductivity; BD: soil bulk density; TN: soil total nitrogen; SOM: soil organic matter; SON: soil organic nitrogen.

Structural equation models (SEMs) were used to evaluate the direct and indirect effects of straw and tillage method on soil abiotic factors (pH, EC, BD, SOM and SON fractions) and biotic factors (bacterial richness and Shannon diversity), as well as their subsequent contributions to soil TN accumulation (Figure 9). The SEM results revealed that soil N accumulation in coastal saline–alkali land, was significantly influenced by straw returning and tillage directly or indirectly. In 2021, bacteria richness was negatively affected by EC, BD, and SON, and the Shannon diversity of bacteria was profoundly influenced by soil EC, BD. In 2022, bacterial richness was negatively influenced by straw, soil BD, SOM and SON, and bacterial Shannon diversity was positively influenced by pH and SOM. Straw and tillage altered soil’s physical and chemical properties, directly or indirectly, thereby influencing the abundance and diversity of bacteria. However, bacterial abundance and diversity did not directly affect the accumulation of total N. Instead, SON accumulation directly determined soil TN accumulation. Furthermore, the network structure of the model becomes increasingly complex with the extension of experimental period.

## 4. Discussion

### 4.1. Impact on Saline–Alkaline Modification

Tillage practices significantly affected soil EC and BD in 2022, especially deep tillage with straw (Table 1 and Table 2), which is consistent with the previous findings [7], who reported that straw application effectively reduced topsoil salinity. This reduction can be attributed to the fact that deep tillage breaks the plow pan of saline–alkali land, thereby inhibiting upward salt migration [35], while also increasing soil porosity and creating a looser and more permeable soil environment that facilitates water infiltration and salt leaching [7]. However, DTSR significantly reduced soil TN, IN and SON contents compared with STSR in 2022. Straw return significantly influenced soil’s available N (IN, ANR and SON fractions) (Table 1 and Table 2), which is primary attributed to reduced soil salinity, thereby affecting the nitrification rate and promoting the accumulation of NH_4_^+^-N, and the stability of SON fractions such as amino acid N [36,37]. Previous studies have showed that straw return promoted the growth of nitrifying microorganisms, particularly ammonia-oxidizing bacteria and ammonia-oxidizing archaea, thereby accelerating the conversion of NH_4_^+^-N to NO_3_^−^-N, which increases the ANR [38,39]. However, our results contradict these findings, as DTSR reduced SON and its components (Table 2). This discrepancy may arise from the dual regulatory effects of straw return on soil nitrification: it not only regulates nitrification, but also enhances microbial immobilization of NH_4_^+^, reduces the available NH_4_^+^-N to NO_3_^−^-N and ultimately decreases ANR [39,40]. Additionally, deep-layer straw return intercepts N, particularly reducing NO_3_^−^ leaching losses [9]. Furthermore, recalcitrant N components in straw contributed to increased soil N retention [41], supporting our observation of increased acid-insoluble N and hydrolysable unknown N under DTSR in 2022 (Table 2).

### 4.2. Impacts on Bacterial Community Composition and Diversity

As the core driver of biogeochemical cycles, the structure and diversity of the soil bacterial community were significantly modulated by straw return and tillage practices [42]. Our results showed that straw return significantly enriched bacteria taxa involved in SOM degradation and the N transformation, such as *Actinobacteria* and *Gammaproteobacteria* (Figure 5 and Figure 9), and promoted the formation of a stable microbial community structure in 2022 (Figure 6), suggesting the establishment of a mature N cycling microbial network with high functional redundancy. Consistent with previous findings, straw return promoted an increase in soil bacterial species richness, and promoted the gradual stabilization of microbial abundance over time, which was closely dependent on N availability [32,43,44]. In contrast, tillage regimes acted as the predominant driver of bacterial community assembly, as strongly verified through SEM analyses (Figure 6 and Figure 8). Increased tillage depth reduced bacterial species richness and evenness, primarily due to less favorable environmental conditions (e.g., water, temperature, and aeration) in deeper soil layers compared to the surface soil [45]. Notably, subsoiling increased bacterial species richness, possibly because it minimized disturbance to the plow layer, improved water and air permeability in deep soil, enhanced soil aggregates formation, and strengthened the soil structural stability [46].

The Mantel test results further confirmed that bacterial richness was closely correlated with soil properties (Figure 8), with soil pH, SOC and BD serving as key drivers of microbial structure and activity [27]. It has been well documented that soil bacteria mediate soil acidification via organic acid secretion [47], while bacterial ammonification and denitrification are mainly mediated by NH_4_^+^-N, a highly alkaline by-product of protein and amino acid catabolism, which critically modulates soil N transformation and pH homeostasis [18]. In this study, bacterial community structure and diversity are mainly mediated by soil C and N supply and availability (Figure 8). Furthermore, straw return increased the abundance of N-fixed-related bacteria—*Cyanobacteriota* (Figure 5)—which contributes to soil salt tolerance, N fixation, SOM accumulation, soil structure improvement and plant growth promotion [27]. Such microbial alterations were primary attributed to the straw-induced increase in SOM and SON (Table 1 and Table 2), which provided sufficient substrates for microbial growth and reproduction [48], and the improved soil aeration, water retention and nutrition availability further optimized the microenvironment for these taxa [49].

### 4.3. Regulation of Soil N Accumulation by Bacteria

This study confirms that the bacterial community is a key bridge connecting straw return and tillage practices to soil N accumulation. In 2022, DTSR reduced SON, with hydrolysable NH_4_^+^-N, amino acid N, amino sugar N decreasing (Table 2). Meanwhile, the relative abundance of N-fixing-related bacteria including *Cyanobacteriota* and *Pseudomonadota*, significantly increased under DTSR (Figure 4). Decomposing straw can supply exogenous N sources and facilitate the fixation of atmospheric N_2_ into biomass N [50]. This phenomenon supports the microbial N mining hypothesis, which posits that microorganisms activate biological N fixation to meet metabolic demands during the decomposition of high C/N ratio straw [51,52]. In 2022, rotary tillage significantly increased stable SON compared with deep tillage and subsoiling with or without straw (Table 2). RTSR also maintained a higher bacterial Shannon diversity index and a more stable community structure of the top 20 cm soil (Figure 6h). Research showed that community stability can enhance the ecosystem resistance to external disturbances and a more mature N cycling network [53]. This may explain why, under RTSR, despite active N transformation, N is retained in more stable forms (such as SSON) in the soil N pools, reducing the risk of loss. Mantel test indicated that bacterial β-diversity (PC1) was significantly correlated with SOM, ANR, HNN and ASN (Figure 8), highlighting SOM as a key environmental factor driving bacterial community structure. Straw return enhances SOM, providing a superior energy foundation for microorganisms [32]. SEMs further confirmed that tillage and straw return did not directly affect TN accumulation, but acted through the indirect path of “environmental factors–bacterial community–SON” (Figure 9). Specifically, in 2022, practices such as RTSR reduced soil salinity and increased SOM, thereby improving the physical environment and enriching specific functional bacteria (such as *Cyanobacteriia*). The activity of these microorganisms directly determines the content and form of SON, an important intermediate pool for N conversion, which ultimately directly governs soil N accumulation [54].

## 5. Conclusions

This study revealed that straw return combined with tillage effectively improved soil’s physical and chemical conditions and shifted bacterial communities to regulate N retention in coastal saline–alkaline soils of the Yellow River Delta. Subsoiling and deep tillage with straw return (STSR, DTSR) significantly reduced soil bulk density by 7–8%, while rotary tillage with straw return (RTSR) increased soil organic matter by 5–16% and effectively decreased soil salinity. Tillage predominantly governed bacterial α-diversity, and N availability acted as the key factor shaping both α- and β-diversity. SEM verified that SON accumulation directly drove total N accumulation. STSR was identified as the optimal practice for sustaining active SON fractions and enhancing soil N sequestration capacity. These findings supplement the biogeochemical theory of N cycling in saline–alkali lands and provide a scientific basis for regional soil management. For practical application, STSR is recommended for improving soil structure and stabilizing N supply, while rotary tillage with straw return is preferred for mitigating soil salinity. Future research will focus on long-term field experiments and explore fungal communities and N cycling functional genes to reveal the underlying molecular mechanisms.

## Figures and Tables

**Table 1:** Physical and chemical properties of the top 20 cm soil under different straw and tillage method in two years.

Year	Treatment	pH	EC	BD	SOM	TN	SON	ANR
			(us/cm)	(g/cm^3^)	(g/kg)	(g/kg)	(g/kg)	(%)
2021	RTNS	8.3 ± 0.1 a	191.1 ± 5 a	1.5 ± 0.1 ab	17.1 ± 0.8 ab	1.1 ± 0.1 a	1.0 ± 0.1 a	0.9 ± 0.0 a
	RTSR	8.2 ± 0.1 a	191.6 ± 18 a	1.5 ± 0.1 a	18.0 ± 0.5 a	1.2 ± 0.1 a	1.2 ± 0.1 a	0.9 ± 0.0 ab
	DTNS	8.3 ± 0.1 a	182.1 ± 3 a	1.4 ± 0.0 ab	17.0 ± 0.9 ab	1.0 ± 0.1 a	1.0 ± 0.1 a	0.8 ± 0.1 b
	DTSR	8.2 ± 0.1 a	184.5 ± 11 a	1.4 ± 0.0 b	16.5 ± 0.6 bc	1.0 ± 0.1 a	0.9 ± 0.1 b	0.9 ± 0.1 ab
	STNS	8.2 ± 0.2 a	197.2 ± 13 a	1.5 ± 0.1 ab	15.4 ± 0.1 c	1.0 ± 0.2 a	0.9 ± 0.2 ab	0.8 ± 0.1 b
	STSR	8.2 ± 0.1 a	195.7 ± 10 a	1.5 ± 0.0 ab	18.0 ± 0.8 a	1.0 ± 0.3 a	1.0 ± 0.3 ab	0.9 ± 0.0 ab
2022	RTNS	8.3 ± 0.0 b	183.9 ± 6 c	1.4 ± 0.0 a	17.2 ± 0.6 c	1.1 ± 0.0 a	1.1 ± 0.0 a	0.6 ± 0.0 b
	RTSR	8.4 ± 0.1 a	177.3 ± 15 c	1.5 ± 0.1 a	20.0 ± 0.4 a	1.2 ± 0.0 a	1.2 ± 0.0 a	0.7 ± 0.0 ab
	DTNS	8.3 ± 0.1 b	234.5 ± 1 b	1.3 ± 0.1 a	17.8 ± 0.6 bc	1.2 ± 0.0 a	1.1 ± 0.0 a	0.6 ± 0.1 b
	DTSR	8.3 ± 0.0 b	279.0 ± 19 a	1.4 ± 0.1 a	17.4 ± 0.4 c	1.0 ± 0.1 b	1.0 ± 0.1 b	0.6 ± 0.1 b
	STNS	8.4 ± 0.0 ab	217.0 ± 7 b	1.4 ± 0.1 a	17.3 ± 0.0 c	1.1 ± 0.0 a	1.1 ± 0.0 a	0.4 ± 0.1 c
	STSR	8.4 ± 0.1 ab	223.3 ± 10 b	1.4 ± 0.1 a	18.6 ± 0.7 b	1.1 ± 0.0 a	1.1 ± 0.0 a	0.7 ± 0.0 a
Significance	S	0.176	4.127	0.373	32.166 ***	0.007	0.007	23.104 ***
	T	0.481	5.763 **	1.284	5.993 **	1.148	1.245	3.647 *
	Y	15.563 ***	59.148 ***	2.032	28.562 ***	6.115 *	6.981 *	256.362 ***
	S × T	0.521	27.23 ***	0.811	17.708 ***	3.631 *	3.457 *	7.373 **
	S × Y	2.961	3.62	0.003	0.437	0.805	0.915	11.21 **
	T × Y	1.734	12.164 ***	0.18	5.440 *	0.664	0.585	3.584 *
	S × T × Y	0.214	34.143 ***	2.618	0.5225	0.315	0.314	0.68

Note: Values are average ± standard errors (*n* = 3). Means followed by the little letters indicate significant differences among six treatments at the top 20 cm soil layers in 2021 and 2022, respectively (Duncan, *p* < 0.05). RTNS: rotary tillage without straw; DTNS: deep tillage without straw; STNS: subsoiling without straw; RTSR: rotary tillage with straw; DTSR: deep tillage with straw; STSR: Subsoiling with straw. S: straw return method; T: tillage practice; Y: year. * *p* ≤ 0.05, ** *p* ≤ 0.01, *** *p* ≤ 0.001, level of significance (two-tailed) by least significant difference (LSD). EC: electrical conductivity; BD: bulk density; SOM: soil organic matter; TN: total nitrogen; SON: soil organic nitrogen; ANR: apparent nitrogen nitrification rate.

**Table 2:** The concentrations (mg kg^−1^) of SON fractions in top 20 cm soil under different straw and tillage method in 2021 and 2022.

Year	Treatment	AHN	AAN	ASN	HUN	AIN	ASON	SSON
2021	RTNS	284.5 ± 27 ab	35.0 ± 4 c	46.2 ± 6 d	549.0 ± 18 a	186.6 ± 19 b	365.6 ± 31 b	735.6 ± 14 a
	RTSR	303.5 ± 11 a	64.1 ± 7 b	74.6 ± 3 c	562.2 ± 28 a	200.1 ± 2 b	442.2 ± 12 a	762.3 ± 30 a
	DTNS	270.5 ± 6 bc	80.1 ± 8 a	79.1 ± 2 bc	440.9 ± 21 c	52.6 ± 20 d	429.6 ± 10 a	493.5 ± 17 d
	DTSR	287.9 ± 3 ab	82.0 ± 12 a	90.1 ± 2 b	430.4 ± 41 c	131.6 ± 22 c	460.0 ± 11 a	562.0 ± 54 c
	STNS	253.8 ± 14 c	72.0 ± 6 ab	117.6 ± 11 a	495.5 ± 24 b	106.6 ± 21 c	443.4 ± 9 a	602.1 ± 19 c
	STSR	272.6 ± 3 bc	85.8 ± 5 a	83.9 ± 6 bc	433.9 ± 7 c	245.4 ± 2 a	442.3 ± 12 a	679.3 ± 5 b
2022	RTNS	320.4 ± 11 ab	42.4 ± 2 a	88.0 ± 1 cd	541.5 ± 10 a	193.5 ± 2 a	450.8 ± 9 bc	735.1 ± 9 b
	RTSR	335.1 ± 13 ab	48.8 ± 6 a	108.3 ± 2 b	555.1 ± 18 a	132.6 ± 7 b	492.1 ± 9 ab	838.8 ± 51 a
	DTNS	307.8 ± 25 ab	44.4 ± 8 a	82.9 ± 12 d	508.4 ± 12 b	175.5 ± 14 a	435.1 ± 30 bc	641.0 ± 17 c
	DTSR	293.6 ± 54 b	13.7 ± 1 c	81.2 ± 6 d	572.0 ± 20 a	283.7 ± 32 a	388.5 ± 58 c	637.4 ± 20 c
	STNS	369.9 ± 46 a	46.7 ± 3 a	123.7 ± 6 b	443.6 ± 25 d	65.4 ± 32 c	540.2 ± 49 a	619.1 ± 37 c
	STSR	335.7 ± 52 ab	25.0 ± 7 b	132.6 ± 24 a	478.3 ± 10 c	108.0 ± 14 b	493.3 ± 70 ab	586.2 ± 16 c
Significance	S	0.773	21.462 ***	3.226	1.518	3.544	0.65	17.89 ***
	T	2.061	8.719 **	31.169 ***	25.108 ***	11.95 ***	10.829 ***	42.696 ***
	Y	26.001 ***	232.469 ***	45.564 ***	18.893 ***	35.158 ***	10.604 **	15.452 ***
	S × T	2.135	21.48 ***	32.213 ***	32.387 ***	49.54 ***	2.547	100.85 ***
	S × Y	1.137	5.161 *	1.372	15.753 ***	108.942 ***	5.626 *	3.442
	T × Y	0.692	32.12 ***	22.572 ***	12.182 ***	25.282 ***	6.917 **	29.652 ***
	S × T × Y	0.773	21.462 ***	3.226	1.518	3.544	0.65	17.89 ***

Note: Values are average ± standard errors (*n* = 3). Means followed by the little letters indicate significant differences among six treatments at the top 20 cm soil layers in 2021 and 2022, respectively (Duncan, *p* < 0.05). RTNS: rotary tillage without straw; DTNS: deep tillage without straw; STNS: subsoiling without straw; RTSR: rotary tillage with straw; DTSR: deep tillage with straw; STSR: subsoiling with straw. S: straw return method; T: tillage practice; Y: year. * *p* ≤ 0.05, ** *p* ≤ 0.01, *** *p* ≤ 0.001, level of significance (two-tailed) by least significant difference (LSD). ANH: hydrolysable NH_4_^+^-N; AAN: amino acid N; ASN: amino sugar N; HUN: hydrolysable unknown N; AIN: acid-insoluble N; ASON: active SON; SSON: stable SON.

## Data Availability

All relevant data are contained within the article.
